# Transthoracic Migration of a Foreign Body into the Diaphragm from the Gunshot Injury and Its Management in a Child: A Case Report

**DOI:** 10.1055/s-0042-1756198

**Published:** 2022-09-02

**Authors:** Klein Dantis, Pranay Suresh Mehsare, Subrata Kumar Singha, Nilesh Gupta

**Affiliations:** 1 Department of Cardiothoracic Surgery, All India Institute of Medical Sciences, Raipur, Chhattisgarh, India; 2 Department of Anesthesiology, All India Institute of Medical Sciences, Raipur, Chhattisgarh, India; 3 Department of Radiology, All India Institute of Medical Sciences, Raipur, Chhattisgarh, India

**Keywords:** hemopneumothorax, foreign body, diaphragm, C-arm, minithoracotomy

## Abstract

Intrapleural foreign bodies (FB) are rare and uncommon, while diaphragmatic FB secondary to gunshot injury in a child is still rarer. We now describe a 9-year-old male with a history of self-inflicted accidental air gun injury on the right side of the midline of the sternum with transthoracic migration of FB—lead bullet—measuring 1cm x1.4cm into the diaphragm managed initially with intercostal tube drainage for right hemopneumothorax at the different center underwent thoracoscopy followed by minithoracotomy and retrieval under C-arm guidance that has not been reported in the literature.

Intrapleural foreign bodies (FB) are rare and uncommon, while diaphragmatic FB is still rarer. 1 Nevertheless, removing a diaphragmatic FB is a challenge, when visualized or palpable; however, undetected minute particles measuring 1 cm undetected or not palpable always pose a therapeutic challenge.

## Case Report

**Fig. 1:**
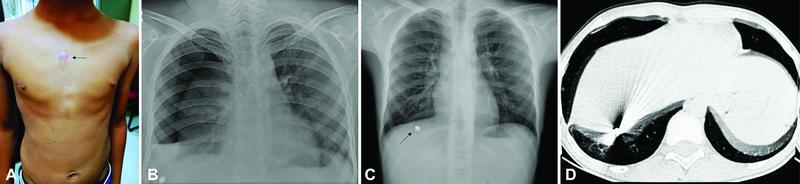
( **A** ) Wound of entry at the right side of the midline of the sternum. ( **B** ) Chest X-ray showing right-sided hydropneumothorax with a homogenous opacity in the right hemidiaphragm. ( **C** ) Chest X-ray showing completely expanded lung with a homogenous opacity in the right hemidiaphragm after 1 month of initial treatment. ( **D** ) Computed tomography showing multiple casting streak artifacts suggestive of high-density object measuring 1×1.4 cm.

A 9-year-old male with a history of self-inflicted accidental air gun injury 1 month back to the anterior chest wall presented with a wound of entry just to the right of the midline in the second intercostal space ( Fig. 1A ). A month ago, self-inflicted injury leading to dyspnea as a consequence of right hemopneumothorax was managed with intercostal tube drainage (ICD) at different center and simultaneously; he was diagnosed to have homogenous opacity in the right diaphragm suggestive of a FB—air-rifle bullet—( Fig. 1B ) for which he was referred to a higher center.

On presentation, he was asymptomatic and hemodynamically stable with a completely expanded right lung ( Fig. 1C ). Computed tomography (CT) axial section of the lung window detected a high-density object in the right hemidiaphragm measuring 1cm x1.4cm casting plenty of streak artifacts with no pneumothorax ( Fig. 1D ). He underwent thoracoscopy followed by minithoracotomy and removal of a FB within the diaphragm under single lung ventilation.

**Fig. 2:**
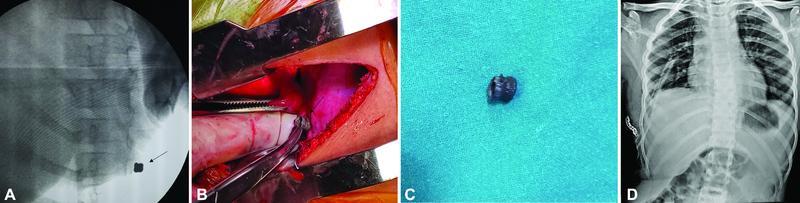
( **A** ) C-arm detecting metallic foreign body (FB) that was undetected on thoracoscopy. ( **B** ) Intraoperative image following removal of an embedded FB. ( **C** ) Metallic FB—pellet. ( **D** ) Postoperative chest X-ray day 1 following FB removal.

**Fig. 3:**
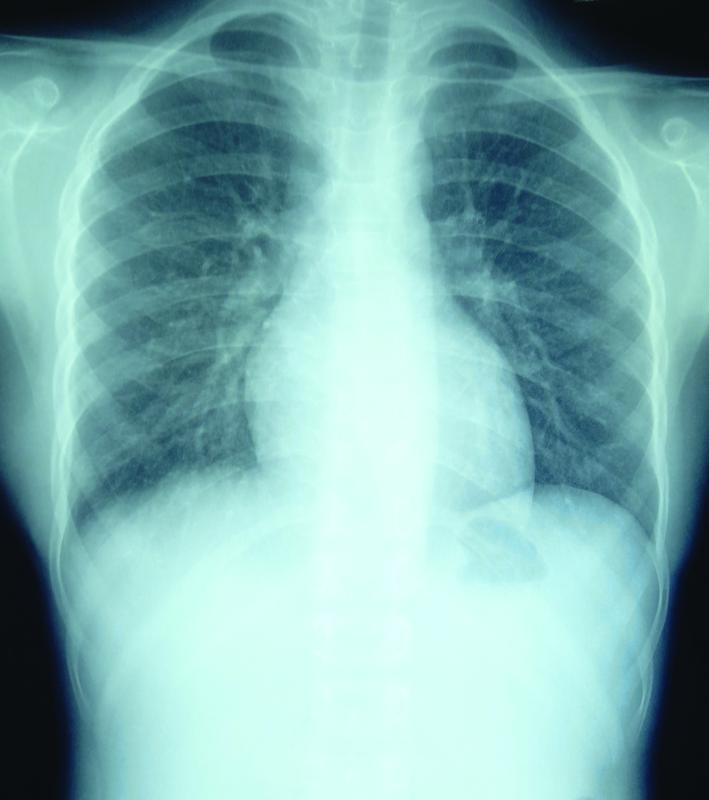
Posteroanterior view of chest X-ray at sixth month follow-up period.

A 2cm incision was performed in the seventh intercostal space in the posterior axillary line. The incision site was decided based on the chest ultrasonogram performed by the anesthetist for FB prior to incision. On thoracoscopy, FB was not visible over the right hemidiaphragm; however, adhesions present between the base of the lung parenchyma and diaphragm were excised. Hence, intraoperatively C-arm was used to detect a metallic FB ( Fig. 2A ). A 2cm incision was extended posteriorly to 5cm for better instrumentation, the pediatric chest retractor was placed, and the metallic FB was traced under C-arm guidance. The diaphragm at the site of embedded FB was opened with cautery, held with Babcock forceps and retrieved, and the site was cleaned with betadine sponges, and the defect was closed with Prolene 3–0 interrupted sutures ( Fig. 2B, C ). The chest cavity was sterilized with a mixture of betadine and hydrogen peroxide in the ratio of 4:1, followed by warm saline washes. A single drain was placed through the separate incision site with well-expanded lung and no detected air-leak intraoperatively. The postoperative course was uneventful, with good lung expansion on day 1 ( Fig. 2D ), a drain removed on day 3, and the patient discharged on day 4. Postoperative follow-up at the third and sixth months ( Fig. 3 ) was uneventful.

## Discussion

Penetrating traumatic injury accounts for 10 to 20% of pediatric trauma and has a significantly higher mortality rate. 2 Historically, air guns were considered powerful and lethal weapons. Ktsebias II, in around 250 BC, first described the use of compressed air to propel a projectile. 3 Later in the 15th century, air guns known as wind chambers were designed using an air reservoir connected to a cannon barrel. 3 With this device, a four-pound lead could propel a distance of 500 yards and penetrate up to a 3-inch oak board. 3 Then in the 17th and 18th centuries, during Napoleon wars, with their ability to fire 15 to 20 rounds a minute and some able to kill 500 lbs stag at a range of 150 paces, air guns were considered firearms. 3 4

The definition of a modern air gun varies from country to country. In the United Kingdom, air pistols with muzzle energy of more than 8.1J and air rifles generating energy of more than 16.2J are termed as “specifically dangerous firearms” and require possession of a Firearm Certificate. 3 4 In contrast, those below this energy level do not require a license; hence, they are purchased by anyone above 18. In addition, in the United Kingdom, children below 14 years can still fire an air gun without any supervision on private land and under the supervision of 21 years and above. 4 In Sweden, as per the Weapons Act, 1996:67, air guns, considered firearms, require a license unless supervised by an adult. In India, as per Schedule I, Category III, Clause “f” of Arms Rule Act 2016, air weapons are classified into two categories. More than 20 Joules muzzle energy or bore exceeding more than 4.5mm under Category III (f) (i) requires a special license for possession and usage. In contrast, weapons with muzzle energy less than 20 Joules or bore below 4.5mm under Category III (f) (ii) do not require a license.

Air guns considered toys have accidentally led to life-threatening injuries, with lead pellets being the most common projectile, similar to our case. 3 Most injuries occur in children and adolescents, with the head and neck region being the most common site. Injuries to the head and neck, eyes, genitourinary area, and abdomen, including appendix and pancreas, have been published with no reports to date with diaphragmatic FB. 2 4

The migration mechanism is explained as follows—FB penetrated through the second intercostal space of the right side, entered the pleural cavity, caused parenchymal injury manifesting as hemopneumothorax, gravitated into the bottom of right pleural cavity caused by the movement of lung and chest wall, and further moved medially on the right hemidiaphragm due to the diaphragmatic movements of respiration. Gradually, its sharp margin got embedded into the diaphragm, not leading to any catastrophe. The prolonged contact of the FB with the diaphragm, helped by the pressure gradient between the thorax and abdomen, can lead to progressive ischemia of diaphragmatic muscle fibers and their erosion, resulting in fistulization into the abdominal cavity, which was not the scenario present in our case. 5

The clinical manifestation is variable. As FB interfered with normal pleuroparenchymal mechanics, patient developed dyspnea, manifesting as hemopneumothorax, which alleviated on ICD insertion. CT is the investigation of choice. 5 6 CT is useful in localizing the site of the FB in the pleural cavity, mediastinum, or pulmonary parenchyma, planning the surgical procedure and identifying the type of the FB, associated abscess or granuloma, and the presence or absence of radiopaque wire markers. 6 As FB was metallic, characteristic casting streaks artifacts were visible on CT.

Management remains a challenge and requires rapid intervention as these FBs are tiny and undetected and can penetrate into the tissue planes, leading to possible intrathoracic/intraabdominal organ injury. In our case, thoracoscopy under high magnification was performed to localize the FB, decide the incision site, and for a better instrumentation approach to retrieve the FB; however, we could not visualize, due to the deeper penetration into the diaphragmatic muscle. Hence, C-arm guidance was required to localize the FB intraoperatively followed by a minithoracotomy. Retrieval of tiny embedded diaphragmatic FB again is still a challenge because of possible penetration further into the abdominal cavity or intraabdominal organ injury. We extended the incision by 3cm posteriorly, limiting it to 5cm, and placed a pediatric chest retractor for better instrumentation to successfully retrieve a FB. Sterilizing the thoracic cavity with an antibiotic or antiseptic solution was necessary to prevent postoperative mediastinitis or empyema.

Though recent legislation has minimized the age for the usage of air guns in public places, we believe stricter regulations need to be implemented by the government to reduce air gun-related injuries. 7 Mass media should bring awareness and educate people on life-threatening sequelae. Every person should consider “self-caring responsibility” an essential aspect of life.

## Conclusion

Embedded, tiny diaphragmatic FBs with normal-appearing diaphragmatic surfaces always pose a therapeutic challenge and require proper preoperative and intraoperative planning by the team of surgeons, radiologists, and anesthetists for the successful outcome.
